# The Long Journey of Pollen Tube in the Pistil

**DOI:** 10.3390/ijms19113529

**Published:** 2018-11-09

**Authors:** Yang-Yang Zheng, Xian-Ju Lin, Hui-Min Liang, Fang-Fei Wang, Li-Yu Chen

**Affiliations:** 1 Fujian Provincial Key Laboratory of Haixia Applied Plant Systems Biology, Key Laboratory of Ministry of Education for Genetics, Breeding and Multiple Utilization of Crops, Center for Genomics and Biotechnology, School of Life Sciences, Fujian Agriculture and Forestry University, Fuzhou 350002, China; zhengyangyang12345@163.com (Y.-Y.Z.); linxianju0825@163.com (X.-J.L.); lhuimin1993@163.com (H.-M.L.); 2 State Key Laboratory of Ecological Pest Control for Fujian and Taiwan Crops, College of Plant Protection, Fujian Agriculture and Forestry University, Fuzhou 350002, China; stephaniefangfei@yahoo.com

**Keywords:** female gametophyte, male gametophyte, pollen tube guidance, sexual reproduction, stigma, transmitting tract

## Abstract

In non-cleistogamous plants, the male gametophyte, the pollen grain is immotile and exploits various agents, such as pollinators, wind, and even water, to arrive to a receptive stigma. The complex process of pollination involves a tubular structure, i.e., the pollen tube, which delivers the two sperm cells to the female gametophyte to enable double fertilization. The pollen tube has to penetrate the stigma, grow in the style tissues, pass through the septum, grow along the funiculus, and navigate to the micropyle of the ovule. It is a long journey for the pollen tube and its two sperm cells before they meet the female gametophyte, and it requires very accurate regulation to perform successful fertilization. In this review, we update the knowledge of molecular dialogues of pollen-pistil interaction, especially the progress of pollen tube activation and guidance, and give perspectives for future research.

## 1. Introduction

**Figure 1:**
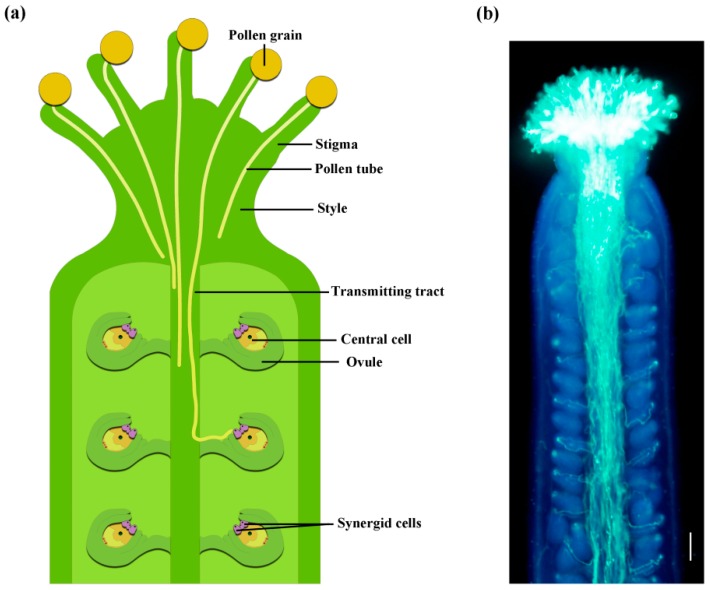
Pollen tube growth in the pistil. (**a**) Schematic diagram of pollen grains that land on the stigma and germinate to produce pollen tubes that grow within the pistil. (**b**) *Arabidopsis thaliana* pollen tubes in a pistil, stained by aniline blue. The image was observed under Olympus BX63 automatic fluorescence microscope. Scale bar = 100 μm.

Successful pollination and fertilization are crucial for sexual plant reproduction in flowering plants. The entire pollination and fertilization process consists of a number of successive steps initiated after pollen landing on the stigma, and its adhesion, hydration, and germination to produce a pollen tube. The pollen tube grows through the style, and then enters into the transmitting tract. It will be attracted by the signals from the ovule [1], and emerge onto the septum, and grow along the funiculus, navigating to the micropyle of the ovule (Figure 1). During pollen tube growth, sperm cells move within the pollen tube, and once the pollen tube reaches the female gametophyte, the two sperms will be released and fuse with the egg cell and the central cell, respectively, for double fertilization. It is a long journey for the pollen tube and its valuable passengers, two sperm cells. There are lots of cell-cell signaling and other interactions involved in these complicated processes. In the past three decades, we have progressed in our understanding of the molecular regulation of the pollen tube journey following the huge advances in plant genetics, genomics, and molecular biology, as well as following the use of model plants. Here, we review the progress of molecular regulation of pollen-pistil interaction.

## 2. Pollen Adhesion and Recognition

Once the pollen grains, released from the anther, arrive to the stigma’s surface, they form productive contacts with the pistil tissues. Different from the intercellular contacts that participate in animal cells, these interactions take place between cells with cell walls and with their extracellular matrices. The mature pollen cell wall includes three main layers with some variation between different species: (1) A pollen coat that fills the empty cavities of the exine, and there are aromatic compounds, lipids, pigments, and proteins within it; (2) an outer strata (exine wall), which is multilayered, composed of sporopollenin, and broken by gaps called apertures; (3) an internal strata (the intine) primarily made of pectin and cellulose [2]. On the basis of the character of the extracellular matrix that covers their surface, stigmas are generally divided into two categories: Wet stigmas coated with viscous secretions are found in various families, including Leguminosae, Solanaceae, and Orchidaceae, and pollen tends to be captured and hydrate nonspecifically on wet stigmas; dry stigmas found in families, such as Asteraceae, Gramineae, and Brassicaceae, are coated with a proteinic pellicle [3]. Because of the surface barriers responsible for hampering pathogen infections, dry stigmas tightly regulate the adopting of pollen [3]. In the Brassicaceae, which includes the model plant, *Arabidopsis thaliana*, dry stigmas are coated with papillae cells, which act as the first point of contact with pollen during pollen-pistil interactions.

Self-incompatibility (SI) is one of the most important systems to preventing inbreeding in many flowering plants. Based on genetic studies, SI can be classified into two systems, gametophytic SI (GSI) and sporophytic SI (SSI), which are distinguished by the genetic behavior of the pollen’s SI phenotype [4]. In the Brassicaceae, SSI will occur rapidly during pollen adhesion to the stigma [5].

On account of the variety of pollen coats and stigma exudates, the pollen-stigma interface is also highly variable. In self-compatible *Arabidopsis*, it is proven that the property of the pollen-stigma interface alters during pollination progresses, becoming fairly tougher over time, with different kinds of adhesive interactions supplanting and replenishing each other [6]. Pollen grains adhere to their own stigma with high affinity; while they bind stigmas from other botanic families poorly, even stigmas from relevant *Brassica* species, indicating a species-specific manner that lower improper pollen access [6]. A very rapid “original” adhesion step was measured that depends on the pollen exine, but not on the pollen coat [6]. The pollen coat is mobilized after exine-mediated adhesion to make the mixture of proteins and lipids extrude on the stigma to form a “pollen foot” [7]. The pollen coat is crucial to successful pollen contact with the dry stigma, and exchange of signals is allowed by the “pollen foot” that results in the activation of the basic compatible pollen responses.

## 3. Pollen Hydration and Germination

Pollen grains need to absorb water from the stigma for hydration and germination. When released from the anther, most mature pollen grains are metabolically inactive and are quite desiccated, and their water content ranges from 15 to 35% [8,9]. With regard to pollen germination and pollen tube growth, the hydration of the dehydrated pollen grains on the stigma surface is a pivotal step. During pollen hydration, the stigma functions as a source of water. Nevertheless, the interaction between pollen and stigma regulates water absorption, particularly on the surface of dry stigma [5,10,11]. The hydration of pollen grains on dry stigma is greatly regulated, as a point that control for rejecting self-pollen grains in the self-incompatible species of the Brassicaceae family [12,13] and for rejecting foreign pollen grains in interspecific crosses [14]. In *Arabidopsis*, it is proved that lipids and proteins from the pollen coat and stigma play a pivotal role in pollen hydration [15]. Previous studies discovered that functional aquaporins are expressed in stigma papillae cells of *Brassica oleracea* [16] and in pollen grains in *Arabidopsis* [17]. In *Arabidopsis*, there are long- and short-chain lipids as well as a small set of proteins existing in the pollen coat, including six lipases and six glycine-rich proteins (GRPs) [18,19]. Mutations in *GRP17* detain the oncoming of pollen hydration and lessen the ability of the mutant pollens to compete with wild-type pollen [20]. Pollens from *eceriferum* (*cer*) mutants, which are defective in long-chain lipids biosynthesis, fail to hydrate on the stigma [13,21]. Interestingly, the defects can be overcome under high humidity, or the addition of wild-type pollen or triacylglycerides to the stigma [21,22,23]. As for mutants in *fiddlehead* lacking a β-ketoacyl-CoA synthase, which is involved in long-chain fatty acids synthesis, the permeability of the leaf cuticle increases and pollen unexpectedly hydrates on inappropriate cell surfaces [24]. The above results provide us a conceivable model that lipids from the male and female surface regulate water transfer from the stigma to pollen, while various proteins modulate self and foreign pollen recognition.

Besides the external components, the internal signaling pathways of pollen also contribute to its hydration. In *Arabidopsis*, the KINβγ subunit of the Snf1-related protein kinase 1 (SnRK1) complex plays an important role in pollen hydration by regulating reactive oxygen species (ROS) levels [25]. The *Arabidopsis kinβγ* mutant pollen grain only has a germination problem on the stigma, while in vitro pollen germination is not affected, indicating that pollen ROS signaling may modulate the water permeability of the stigma cuticle [25]. Further study demonstrated that the KINβγ subunit of the SnRK1 complex can also regulate the expression of *Shaker Pollen Inward K^+^ channel* (*SPIK*) [26].

The perception of mechanical force at the membrane is thought to be critical for pollen hydration. Mechanosensitive (MS) ion channels are adopted to monitor the osmotic challenges and other mechanical stimuli [27]. Recently, a pollen-specific mechanosensitive channel of small conductance like protein, MSL8, was reported to be critical for pollen hydration and germination [28]. Further study proved that ion transport activity of MSL8 is required for its physiological functions [29].

Within minutes after hydration, the pollen grain transforms from nonpolar to highly polarized and organizes its cytoplasm and cytoskeleton to extend a tubular structure. At the same time, the pollen plasma membrane selects targeting secretory vesicle and callose depositing at the site of pollen tube emergence [30]. In many species, the pollen tube will emerge through the pollen aperture, where the pollen wall exine deposition is reduced [31]. Modification of pectin is critical for pollen germination, and knockdown of *Pectin Methylesterase48* (*PME48*) in *Arabidopsis* will display a significant delay in pollen germination [32]. Moreover, pollen cell wall proteins also play an important role in pollen germination, and mutations of *Leucine-Rich Repeat Extensins* (*LRXs*) will lead to compromised pollen germination [33].

Beneath the potential germination site, a Ca^2+^ gradient will be established once the pollen grain is hydrated, which is essential for pollen germination [34]. Mutations of the plasma membrane-localized Ca^2+^ pump [35], Ca^2+^ channel [36], or even the mitochondria-localized Ca^2+^ uniporter complex [37] will cause pollen germination defects. Furthermore, downstream signaling components of Ca^2+^, such as calmodulin [38] and calmodulin-like protein [39], are also involved in pollen germination.

The actin cytoskeleton is involved in the transport of secretory vesicles for cell elongation. Thus, it is also essential for pollen germination. In *Arabidopsis*, actin is encoded by eight functional actin (*ACT*) genes, and mutation of *ACT11* will cause a delay of pollen germination [40]. Actin dynamics are modulated by multiple actin-binding proteins (ABPs), including nucleation factors, depolymerization factors, and filament bundling proteins [41]. Formins act as actin nucleation factors. Recently, a formin family protein, AtFH5, was proved to be essential for polarity establishment and vesicle mobility during pollen germination [42]. Vesicle-localized AtFH5 rotates ahead of actin filaments during pollen germination, and it will translocate to the plasma membrane to initiate the collar-like actin structure at the potential germination site [42]. Actin-depolymerizing factors (ADFs) mainly contribute to actin turnover by depolymerizing actin filaments. Loss-of-function of *ADF5*, a member of subclass III *ADFs*, will lead to delayed pollen germination [43]. Villins and fimbrins are actin filament-bundling factors. Overexpression of a lily (*Lilium longiflorum*) ser/argine-rich (SR) protein in *Arabidopsis* will inhibit pollen germination, while mutation of the *SR45* gene in *Arabidopsis* will lead to earlier pollen germination than that of wild-type [44]. Full-length transcript of *Arabidopsis Villin1* (*AtVLN1*) was increased and the truncated mRNA was decreased in an *SR45* mutant [44]. The expression levels of other *ABPs* were also changed in *SR45* mutant [44]. Loss-of-function of *Arabidopsis FIMBRIN5* (*FIM5*) will also result in delayed pollen germination [45,46].

## 4. Pollen Tube Penetration the Style and Its Interaction with the Sporophytic Tissues

After germination, the pollen tubes have to penetrate the physical barrier of the stigma and the style tissues and extend until they get to the ovule. The genetic regulation of pollen tube penetration remains unclear. Recently, a study reported the identification of *O-Fucosyltransferase1* (*AtOFT1*), a novel *Arabidopsis* gene, participating in pollen tube penetration through the stigma-style interface [47]. AtOFT1 is a Golgi-localized protein, indicating the role of *O*-glycosylation in pollen-pistil interaction [47]. 

There are various substances existing in the extracellular matrix of the transmitting tract that reject non-self pollen tubes in S-RNase-based GSI species [48] or induce pollen tube growth, adhesion, and guidance to the ovary (preovular guidance) in SC (self-compatibility) species [49,50,51]. Pistil-secreted S-RNases are the key barrier proteins in SI in Solanaceae species, and the SLF (S-locus F-box) proteins are pollen resistance factors [48]. The loss-function-of the pistil-side barrier will result in SI transition to SC [48]. Adhesion may be a critical event in intercellular communication in plants. After cryofixation of pollinated styles, the adhesion of pollen tubes to the transmitting tract’s epidermis of the style in some species, including lily and *Arabidopsis*, can be clearly observed [52,53,54,55]. Pollen tubes grown in vivo also adhere to each other, a case not being observed in vitro [53]. Adhesion is necessary for the successful delivery of the pollen tube to the ovary. From an in vitro adhesion assay, two molecules were isolated from the stigma/stylar transmitting tract extracellular matrices of lily that are necessary for pollen tube adhesion [56]: A small (9 kDa) stigma/stylar cysteine-rich adhesin (SCA), which shares similarity with plant lipid transfer proteins (LTPs); a large pectic polysaccharide [55]. SCA associated with pectin was first depicted as an extracellular “glue” for pollen tubes [55,56]. When acting along with a blue copper protein of the plantacyanin family, which is named chemocyanin, SCA also took part in pollen tube guidance as well [57]. Furthermore, it has been shown that SCA produced in the pistil enters the pollen tube tip in an endocytotic manner [58], and possibly functions as a signal for pollen tube tip growth [59]. By using a gain-of-function mutant (*ltp5-1*) for *Arabidopsis* LPT5, an SCA homologue, the biological function of plant LTPs in compatible pollination was further studied [60]. *LTP5* was present in both pollen and the pistil transmitting tract, involving tip growth of pollen tubes and seed development [61]. In *Solanaceous* species, arabinogalactan proteins (AGPs) also participate in pollen tube growth regulation. For example, a tobacco (*Nicotiana tabacum*) transmitting tissue-specific (TTS) AGP functions as a signal guiding pollen tube growth towards the ovary and involves in setting up normal growth rates [62,63]. Besides the molecules from pistil, the extensin-like Pex proteins from pollen may also participate in adhesion when pollen tube grows through the pistil [64].

There are 20 ionotropic *Glutamate Receptor-Like* (*GLR*) genes in the *Arabidopsis* genome, and six *GLRs* are expressed in pollen [65]. GLRs are involved in Ca^2+^ signaling in the pollen tube by controlling [Ca^2+^]_cyt_ through Ca^2+^ influxes in the tip of the pollen tube and affect its growth. Single knockout of *GLR1.2* or *GLR3.7* results in slower pollen tube growth and decreases seed set per silique, supporting a specific role of *GLR1.2* and *GLR3.7* in pollen tube growth [65]. It is D-Ser, which is present in pistil tissues, activating the GLR1.2 Ca^2+^ channel, and regulating pollen tube growth [65]. Another amino acid, γ-aminobutyric acid (GABA), coupling with the Ca^2+^ channel regulates pollen tube growth [66]. GABA is also thought to be involved in pollen tube guidance by a gradient manner, but pollen tube attraction activity has not been observed yet [67]. 

The events that pollen tube growth and guidance are thought to require extracellular cues, which can be transduced to the pollen cytoplasm to promote the cytoskeletal changes and other cytoplasmic events involved in tip growth [68]. How can these cues be perceived and transduced by pollen tubes? Receptor-like kinases (RLKs) are plausible candidates for this function. The pollen-specific RLKs LePRK1 and LePRK2 from tomato (*Solanum lycopersicum*, formerly *Lycopersicon esculentum*) are thought to play roles in pollination and pollen tube growth [69,70,71,72]. These kinases are localized at the plasma membrane of pollen tubes where they form a high molecular weight complex [73]. These receptors involved in pollen tube growth and guidance are not independent, some of which interact with ligands, particularly cysteine-rich protein (CRP) ligands. CRPs are derived from precursors that have a molecular mass ranging from 4 to 16 kD, and contain four to 16 cysteine residues [74]. A pollen-specific cysteine-rich extracellular protein, LAT52 [75], is involved in pollen germination in vitro [76]. LAT52 interacts with the extracellular domain (ECD) of LePRK2 before pollen germination [77]. After pollen germination, LeSTIG1, a CRP from the stigma, interacts with ECD of LePRK1 and LePRK2, forming STIG1-LePRK1 or STIG1-LePRK2 signaling cascades, and facilitating pollen tube growth [78]. In *STIG1* RNA interference (RNAi) plants, the average pollen tube length in transgenic pistils was shorter than in wild-type pistils [72]. In recent years, a plethora of new studies on RLKs demonstrate that ROP (Rho-like small GTPase from plant) signaling pathways may act downstream of them [79]. Kinase partner protein (KPP) [80], a pollen cytoplasmic protein, which was demonstrated to be an ROPGEF (ROP GDP/GTP exchange factor) [81], interacts with LePRK1 and LePRK2. This interaction indicates that there is a connection between extracellular signals, RLKs, and regulation of ROP activities, which is extremely crucial for pollen tube growth [82]. STIL, a peculiar molecule from tobacco styles, specifically dephosphorylates LePRK2 and facilitates pollen tube growth from the beginning of germination in a dose-dependent way [83]. In *Arabidopsis*, PRKs have also been implicated as candidate regulators for perceiving growth-promoting factors [84]. Therefore, PRKs probably act as bridges, which transduce signals from the extracellular environment into the pollen cytoplasm by interacting with specific cytoplasmic components. 

During pollen tube growth in the pistil, it has to penetrate different pistil tissues. Turgor pressure is supposed to play an important role in this process. However, the study of this aspect is rare, and there is no direct evidence from in vivo data to show the importance of turgor pressure regulation. Recently, TurgOr regulation Defect 1 (TOD1) was identified as a turgor pressure regulator during pollen tube growth in vivo [85]. *TOD1* encodes an alkaline ceramidase, which can catalyze ceramide into sphingosine and fatty acid. Phosphorylated sphingosine, sphingosine-1-phosphate (S1P), is a signaling molecule, which has been demonstrated playing a role in stomata movement [86,87]. *TOD1* mutant pollen tubes generate higher turgor pressure than the wild-type pollen tubes, which may affect the building up of the pollen tube wall strength. Consistently, *TOD1* mutant pollen tube growth retardation can be rescued by additional mutation of *Galacturonosyltransferase 13 (GAUT13*), which is involved in pectin biosynthesis in pollen tubes [88]. It suggests that turgor pressure regulation during pollen tube growth in the pistil is vital for successful fertilization.

How can the pollen tube keep its integrity during tube growth? Recently, two independent studies reported the role of CRPs and RLKs in this process [89,90,91]. CRPs, including rapid alkalinization factor (RALFs), act as extracellular signaling ligands, interacting with receptor-like kinases of the *Catharanthus roseus* RLK1-like (CrRLK1L) subclass. There are 36 RALF members in *Arabidopsis* [92]. *RALF4*/*19* are expressed in the mature pollen grains and pollen tubes, and they act redundantly in pollen tube integrity and growth regulation. RALF4/19’s function depends on LRXs, which are pollen tube-expressed proteins and play a role in cell wall development [93]. In artificial microRNA (amiRNA) *RALF4*/*19* transgenic lines, reduced *RALF4*/*19* expression causes only 3.5% of pollen tubes reaching the ovules, and nearly 70% of in vitro germinated pollen tubes burst. *ralf4*, a T-DNA insertion mutant, also showed that nearly 49% of pollen tubes burst. RALFs interact with LRXs, monitoring pollen tube wall integrity [90]. RALF4/19 are also identified as ligands of receptors, Buddha’s Paper Seal 1 (BUPS1) and BUPS2, in *Arabidopsis* [89]. Moreover, BUPS1/2 interacting with receptors, ANXUR1 (ANX1) and ANX2, form BUPS1/2-ANX1/2 sets, which can bind RALF4/19. Thus, RALF4/19 peptides interacting with LRXs and ANX1/2-BUPS1/2 maintain pollen tube integrity during its growth.

## 5. Ovular Pollen Tube Guidance

The female gametophyte of a flowering plant is deeply embedded in the ovule, which is located inside of the ovary. How can pollen tubes in the transmitting tract be precisely guided to the ovule? It is controlled by an ovular pollen tube guidance system, which can be divided into two stages: Funicular guidance, guidance from the surface of the placenta to the funiculus; micropylar guidance, guidance from the entrance of the micropyle to the female gametophyte [94,95]. Ovular pollen tube guidance requires accurate perception of ovule-emitted guidance cues or signals by the receptors in pollen tubes [1,96]. Recently, key proteins and molecules involved in this process have been identified (Table 1) [1,97,98].

Pollen tubes germinated on a simple growth medium cannot be guided to the micropyle of the ovule [113], while pollen tubes germinated semi-in vitro (SIV) (on stigma and through the style) can navigate to the ovule, suggesting that stigma and style tissues prime pollen tubes to respond to female attraction signals [114,115]. A recent study on pollen tube guidance reported that SIV pollen tubes are not competent to respond to attraction signals when they just pass through the cut style, and the ovary originated signal AMOR (activation molecule for response capability), an arabinogalactan sugar chain, induces pollen tube competency in *Torenia fournieri* [99]. Moreover, the growth through the style cannot be fully replaced by adding high concentrations of AMOR, indicating that additional signals exist in the style, contributing to activation of the pollen tube [99]. 

Studies of in vitro pollen tube growth have highlighted the essential roles of ions and pH dynamics in tip growth, indicating the importance of active and spatially localized ion transporters. For example, two ER (endoplasmic reticulum)-localized cation/proton exchangers (CHX), CHX21 and CHX23, which function as K^+^ transporters, are important for pollen tube guidance in *Arabidopsis* [107]. *chx21 chx23* double mutant pollen grains germinate and grow tubes down into the transmitting tract, but the pollen tubes fail to navigate to the funiculus [107]. The Ca^2+^ gradient and oscillation in the pollen tube are also essential for pollen tube growth and guidance, and, therefore, Ca^2+^ channels on the plasma membrane of pollen tubes may play an important role in pollen tube guidance. There are eight Ca^2+^ channels present in pollen tubes, and cyclic nucleotide-gated channel 18 (CNGC18) is the only one critical for pollen tube guidance [108].

The maturity of the female gametophyte will also affect the ovular pollen tube guidance. In *magatama* (*maa*) mutants of *Arabidopsis*, female gametophyte development is delayed, and they show defects in micropylar pollen tube guidance [116]. The mutant pollen tubes can grow onto the funiculus, but later they grow in random directions and lose their way to enter the micropyle. Moreover, the high frequency of two pollen tubes simultaneously attracted by the mutant female gametophytes also occurs. These results indicate that the female gametophytes not only attract pollen tubes, but also prevent multiple pollen tubes into the ovule to prevent polyspermy [116]. Interestingly, GABA also plays a central role in the prevention of multiple pollen tubes targeting a single ovule, and increases in GABA may mask other signals essential for the inhibition of multiple pollen tubes on an ovule [67]. Further study on *MAA3* demonstrated that it encodes a homolog of yeast Sen1 helicase, and is required for fusion of polar nuclei [106]. 

Laser ablation of synergid cells proved that they are essential for the secretion of attractant signals in *Torenia fournieri* [113]. Further study proved that the defensin-like (DEFL) superfamily of CRPs, named LUREs, are secreted as pollen tube attractants from synergid cells [101]. Two *LURE* genes (*TfLURE1* and *TfLURE2*) have been identified in *Torenia fournieri*, which are secreted to the surface of the egg apparatus. In the SIV *Torenia* system, pollen tubes can be attracted by TfLURE1 and TfLURE2, respectively [101,117]. Furthermore, six duplicated *LURE1* genes have been identified in *Arabidopsis* [102]. They also act as attractants, guiding pollen tubes to the micropyle. Interestingly, heterologously expressed AtLURE1 in synergid cells of *Torenia fournieri* was sufficient to attract *Arabidopsis* pollen tubes to a *Torenia fournieri* embryo sac [102]. In the monocot *Zea mays* (maize), another secreted small peptide, the EGG APPARATUS1 (ZmEA1), has been reported to take part in micropylar pollen tube guidance [100,118].

MYB98, an R2R3-type MYB transcription factor expressed in synergid cells, is required for micropylar pollen tube guidance and the formation of the filiform apparatus [103]. In *MYB98* plants, pollen tubes grow normally from the placenta to the funiculus, but then only a few are able to grow into the micropyle, as a result, only 17% of seed sets in *MYB98* mutants [103]. Interestingly, the mutation of *Central Cell Guidance* (*CCG*), a central cell-specific expressed gene, also results in defects in micropylar pollen tube guidance, but it does not affect female gametophyte development [104]. Further study demonstrated that CCG can bind CCG binding protein 1 (CBP1) in central cells to recruit the Mediator complex and RNA Pol II machinery, which may control *LUREs* expression via synergid cell-specific *MYB98* [105]. 

RLKs are also important for regulating ovular pollen tube guidance. Liu et al. discovered that 76 *RLK* genes were preferentially expressed in SIV growth pollen tubes [112]. These pollen-specific *RLKs* were grouped into different subfamilies, several belonging to receptor-like cytoplasmic kinase (RLCK) VII subfamily, such as lost in pollen tube guidance 1 (LIP1) and LIP2 [112]. Simultaneous mutation of *LIP1* and *LIP2* results in impaired pollen tube guidance into the micropyle and significantly reduced attraction of pollen tubes towards the female attractant, AtLURE1 [112]. Liu et al. suggested that LIP1 and 2 represent essential components of the pollen tube receptor complex to perceive the female signal, AtLURE1, for micropylar pollen tube guidance [112]. Since LIP1 and 2 do not have an extracellular domain, whether LIP1 and 2 can interact with LURE1 receptors as co-receptors needs further study. Recently, the receptors of LURE1 were identified by two independent groups [109,110]. MDIS1-MIK1/2, two receptor heteromers, serve as the receptor/co-receptor complexes to perceive the attractant signal, AtLURE1, and transduce the direction signal into the cell by transphosphorylation [109]. The aforementioned PRK family member, PRK6, was also proven to act as the receptor for AtLURE1, and transduce the signal via the ROP signaling cascade [110]. 

According to recent studies, a new receptor-like kinase of the CrRLK1L subfamily, ERULUS (ERU), which is only expressed in pollen and root hairs as a tip growth specific kinase, is involved in pollen tube growth and guidance in *Arabidopsis* [111]. ERU plays a role in regulating apical [Ca^2+^]_cyt_ oscillations in response to [Ca^2+^]_ext_, which help pollen tubes navigating to the ovules [111]. Interestingly, ERU also regulates cell wall composition through pectin methylesterase activity and phosphorylation of FERONIA (FER) and H^+^-ATPase 1/2 during root hair tip growth in *Arabidopsis* [119].

## 6. Pollen Tube Reception and Burst

After the pollen tube enters into the micropyle, it will meet one of two synergid cells. FER, also belonging to the CrRLK1L subfamily, and located in the filiform apparatus of the synergid cells, plays a critical role in pollen tube reception in *Arabidopsis* [120]. Furthermore, female-specific small proteins, early nodulin-like proteins (ENODLs, or ENs), are also involved in pollen tube reception [121]. In *en* mutants, the pollen tube passes the embryo sac, and fails to release its cargo into the embryo sac [121]. EN14 directly and specifically interacts with the extracellular domain of FER [121], and it is closely related to glycosylphosphatidylinositol-anchored proteins (GPI-APs) LORELEI (LRE) and LRE-like GPI-AP1 (LLG1), which also interact with FER [122]. It suggests that these proteins associate with each other and form a large complex to regulate FER-mediated signal for ovule reception.

Once being received, the pollen tube needs to burst to release the two sperm cells. How can the pollen tube trigger tube burst soon after it reaches the female gametophyte? Ge et al. reported that another RALF peptide, RALF34, derived from the ovule and widely distributed around the micropylar/synergid cell region, is able to replace RALF4/19 for binding to BUPS1/2-ANX1/2, demonstrating that RALF34 competes with RALF4/19 when the pollen tube reaches the embryo sac, and then deregulates BUPS1/2-ANX1/2 signaling to promote pollen tube rupture and sperm release [89]. RALF34 also acts as a ligand of THESEUS1 (THE1), a member of the CrRLK1L family [123]. RALF34 and THE1 form RALF34-THE1-mediated signaling to regulate cell wall integrity and lateral root initiation in *Arabidopsis* [123]. In maize, another defensin-like protein, ZmES4 (*Zea mays* embryo sac 4), which is accumulated in the secretory region of the synergid cells, mediates pollen tube rapture by opening of the potassium channel, KZM1 (K^+^ channel *Zea mays*) [124]. In rice, ruptured pollen tube (RUPO), a CrRLK1L subfamily protein, interacts with potassium transporters to regulate the pollen tube integrity [125]. 

## 7. Conclusions and Perspectives

Pollen-pistil interaction is critical for the successful fertilization of flowering plants. The missing activator for pollen tube priming in style tissue in *Arabidopsis* will be of great interest in further studies. More attractant molecules will be discovered with the help of plant ovule secretome [126] combined with transcriptome analysis, which may provide a more delicate map of pollen-pistil interaction. Genetic redundancy is widespread in plant sexual reproduction, which ensures successful fertilization. However, it is a big challenge for plant biologists to unravel the function of these redundant genes, such as *RLKs*. CRISPR/Cas9 technology will be a powerful tool to study the function of these genes, and it is also very useful for small genes, like *CRPs*, in which mutations may be rare even after saturation mutagenesis.

## Figures and Tables

**Table 1:** Key proteins and molecules involved in ovular pollen tube guidance.

Origin	Proteins/Molecules	Ref.
Ovule	AMOR (arabinogalactan polysaccharide)	[99]
Egg apparatus	ZmEA1	[100]
Synergid cells	LUREs	[101,102]
Synergid cells	MYB98	[103]
Central cells	CCG	[104]
Central cells	CBP1	[105]
Undefined	MAA3	[106]
Pollen tube	CHX21 and CHX23	[107]
	CNGC18	[108]
	MDIS1, MIK1 and MIK2	[109]
	PRK6	[110]
	ERU	[111]
	LIP1 and LIP2	[112]

## References

[1] Higashiyama T., Yang W.C. (2017). Gametophytic pollen tube guidance: Attractant peptides, gametic controls, and receptors. Plant Physiol..

[2] Edlund A.F., Swanson R., Preuss D. (2004). Pollen and stigma structure and function: The role of diversity in pollination. Plant Cell.

[3] Swanson R., Edlund A.F., Preuss D. (2004). Species specificity in pollen-pistil interactions. Annu. Rev. Genet..

[4] Iwano M., Takayama S. (2012). Self/non-self discrimination in angiosperm self-incompatibility. Curr. Opin. Plant Biol..

[5] Doucet J., Lee H.K., Goring D.R. (2016). Pollen acceptance or rejection: A tale of two pathways. Trends Plant Sci..

[6] Zinkl G.M., Zwiebel B.I., Grier D.G., Preuss D. (1999). Pollen-stigma adhesion in *Arabidopsis*: A species-specific interaction mediated by lipophilic molecules in the pollen exine. Development.

[7] Elleman C.J., Dickinson H.G. (2010). The role of the exine coating in pollen–stigma interactions in *Brassica oleracea* L.. New Phytol..

[8] Heslop-Harrison J. (1979). An interpretation of the hydrodynamics of pollen. Am. J. Bot..

[9] Buitink J., Leprince O., Hemminga M.A., Hoekstra F.A. (2010). The effects of moisture and temperature on the ageing kinetics of pollen: Interpretation based on cytoplasmic mobility. Plant Cell Environ..

[10] Hiscock S.J., Allen A.M. (2008). Diverse cell signalling pathways regulate pollen-stigma interactions: The search for consensus. New Phytol..

[11] Dresselhaus T., Franklin-Tong N. (2013). Male-female crosstalk during pollen germination, tube growth and guidance, and double Fertilization. Mol. Plant.

[12] Sarker R.H., Elleman C.J., Dickinson H.G. (1988). Control of pollen hydration in *Brassica* requires continued protein synthesis, and glycosylation in necessary for intraspecific incompatibility. Proc. Natl. Acad. Sci. USA.

[13] Hülskamp M., Kopczak S.D., Horejsi T.F., Kihl B.K., Pruitt R.E. (1995). Identification of genes required for pollen-stigma recognition in *Arabidopsis thaliana*. Plant J..

[14] Dickinson H. (1995). Dry stigmas, water and self-incompatibility in *Brassica*. Sex. Plant Reprod..

[15] Murphy D.J. (2006). The extracellular pollen coat in members of the Brassicaceae: Composition, biosynthesis, and functions in pollination. Protoplasma.

[16] Dixit R., Rizzo C., Nasrallah M., Nasrallah J. (2001). The *Brassica MIP-MOD* gene encodes a functional water channel that is expressed in the stigma epidermis. Plant Mol. Biol..

[17] Di Giorgio J.A.P., Bienert G.P., Ayub N.D., Yaneff A., Barberini M.L., Mecchia M.A., Amodeo G., Soto G.C., Muschietti J.P. (2016). Pollen-specific aquaporins NIP4;1 and NIP4;2 are required for pollen development and pollination in *Arabidopsis thaliana*. Plant Cell.

[18] Mayfield J.A., Fiebig A., Johnstone S.E., Preuss D. (2001). Gene families from the *Arabidopsis thaliana* pollen coat proteome. Science.

[19] Fiebig A., Kimport R., Preuss D., Nasrallah J.B. (2004). Comparisons of pollen coat genes across Brassicaceae species reveal rapid evolution by repeat expansion and diversification. Proc. Natl. Acad. Sci. USA.

[20] Mayfield J.A., Preuss D. (2000). Rapid initiation of *Arabidopsis* pollination requires the oleosin-domain protein GRP17. Nat. Cell Biol..

[21] Preuss D., Lemieux B., Yen G., Davis R.W. (1993). A conditional sterile mutation eliminates surface components from *Arabidopsis* pollen and disrupts cell signaling during fertilization. Genes Dev..

[22] Fiebig A., Mayfield J.A., Miley N.L., Chau S., Fischer R.L., Preuss D. (2000). Alterations in *CER6*, a gene identical to *CUT1*, differentially affect long-chain lipid content on the surface of pollen and stems. Plant Cell.

[23] Woltersarts M., Lush W.M., Mariani C. (1998). Lipids are required for directional pollen-tube growth. Nature.

[24] Lolle S.J., Hsu W., Pruitt R.E. (1998). Genetic analysis of organ fusion in *Arabidopsis thaliana*. Genetics.

[25] Gao X.Q., Liu C.Z., Li D.D., Zhao T.T., Li F., Jia X.N., Zhao X.Y., Zhang X.S. (2016). The *Arabidopsis* KINβγ subunit of the SnRK1 complex regulates pollen hydration on the stigma by mediating the level of reactive oxygen species in pollen. PLoS Genet..

[26] Li D.D., Guan H., Li F., Liu C.Z., Dong Y.X., Zhang X.S., Gao X.Q. (2017). *Arabidopsis* shaker pollen inward K^+^ channel SPIK functions in SnRK1 complex-regulated pollen hydration on the stigma. J. Integr. Plant Biol..

[27] Hamilton E.S., Schlegel A.M., Haswell E.S. (2015). United in diversity: Mechanosensitive ion channels in plants. Annu. Rev. Plant Biol..

[28] Hamilton E.S., Jensen G.S., Maksaev G., Katims A., Sherp A.M., Haswell E.S. (2015). Mechanosensitive channel MSL8 regulates osmotic forces during pollen hydration and germination. Science.

[29] Hamilton E.S., Haswell E.S. (2017). The tension-sensitive ion transport activity of MSL8 is critical for its function in pollen hydration and germination. Plant Cell Physiol..

[30] Johnson S.A., McCormick S. (2001). Pollen germinates precociously in the anthers of *raring-to-go*, an *Arabidopsis* gametophytic mutant. Plant Physiol..

[31] Li P., Schuler S.B., Reeder S.H., Wang R., Santiago V.N.S., Dobritsa A.A. (2018). INP1 involvement in pollen aperture formation is evolutionarily conserved and may require species-specific partners. J. Exp. Bot..

[32] Leroux C., Bouton S., Kiefer-Meyer M.C., Fabrice T.N., Mareck A., Guénin S., Fournet F., Ringli C., Pelloux J., Driouich A. (2015). PECTIN METHYLESTERASE48 is involved in *Arabidopsis* pollen grain germination. Plant Physiol..

[33] Wang X.X., Wang K.Y., Yin G.M., Liu X.Y., Liu M., Cao N.N., Duan Y.Z., Gao H., Wang W.L., Ge W.N. (2018). Pollen-expressed leucine-rich repeat extensins are essential for pollen germination and growth. Plant Physiol..

[34] Steinhorst L., Kudla J. (2013). Calcium—A central regulator of pollen germination and tube growth. Biochim. Biophys. Acta Mol. Cell Res..

[35] Li Y., Guo J., Yang Z., Yang D.L. (2018). Plasma membrane-localized calcium pumps and copines coordinately regulate pollen germination and fertility in *Arabidopsis*. Int. J. Mol. Sci..

[36] Gu L.L., Gao Q.F., Wang Y.F. (2017). Cyclic nucleotide-gated channel 18 functions as an essential Ca^2+^ channel for pollen germination and pollen tube growth in *Arabidopsis*. Plant Signal. Behav..

[37] Selles B., Michaud C., Xiong T.C., Leblanc O., Ingouff M. (2018). *Arabidopsis* pollen tube germination and growth depend on the mitochondrial calcium uniporter complex. New Phytol..

[38] Landoni M., De Francesco A., Galbiati M., Tonelli C. (2010). A loss-of-function mutation in *Calmodulin2* gene affects pollen germination in *Arabidopsis thaliana*. Plant Mol. Biol..

[39] Wang S.S., Diao W.Z., Yang X., Qiao Z., Wang M., Acharya B.R., Zhang W. (2015). *Arabidopsis thaliana* CML25 mediates the Ca^2+^ regulation of K^+^ transmembrane trafficking during pollen germination and tube elongation. Plant Cell Environ..

[40] Chang M., Huang S.J. (2015). *Arabidopsis* ACT11 modifies actin turnover to promote pollen germination and maintain the normal rate of tube growth. Plant J..

[41] Staiger C.J., Poulter N.S., Henty J.L., Franklin-Tong V.E., Blanchoin L. (2010). Regulation of actin dynamics by actin-binding proteins in pollen. J. Exp. Bot..

[42] Liu C., Zhang Y., Ren H. (2018). Actin polymerization mediated by AtFH5 directs the polarity establishment and vesicle trafficking for pollen germination in *Arabidopsis*. Mol. Plant.

[43] Zhu J.G., Nan Q., Qin T., Qian D., Mao T.L., Yuan S.J., Wu X.R., Niu Y., Bai Q.F., An L.Z. (2017). Higher-ordered actin structures remodeled by *Arabidopsis* ACTIN-DEPOLYMERIZING FACTOR5 are important for pollen germination and pollen tube growth. Mol. Plant.

[44] Cao L.J., Zhao M.M., Liu C., Dong H.J., Li W.C., Ren H.Y. (2013). LlSR28 is involved in pollen germination by affecting filamentous actin dynamics. Mol. Plant.

[45] Wu Y.J., Yan J., Zhang R.H., Qu X.L., Ren S.L., Chen N.Z., Huang S.J. (2010). *Arabidopsis* FIMBRIN5, an actin bundling factor, is required for pollen germination and pollen tube growth. Plant Cell.

[46] Su H., Feng H.L., Chao X.T., Ding X., Nan Q., Wen C.X., Liu H.D., Xiang Y., Liu W. (2017). Fimbrins 4 and 5 act synergistically during polarized pollen tube growth to ensure fertility in *Arabidopsis*. Plant Cell Physiol..

[47] Smith D.K., Jones D.M., Lau J., Cruz E.R., Brown E., Harper J.F., Wallace I.S. (2018). A putative protein O-fucosyltransferase facilitates pollen tube penetration through the stigma-style interface. Plant Physiol..

[48] Bedinger P.A., Broz A.K., Tovar-Mendez A., McClure B. (2017). Pollen-pistil interactions and their role in mate selection. Plant Physiol..

[49] Taylor L.P., Hepler P.K. (1997). Pollen germination and tube growth. Annu. Rev. Plant Physiol. Plant Mol. Biol..

[50] Cheung A.Y. (1996). Pollen-pistil interactions during pollen-tube growth. Trends Plant Sci..

[51] Luu D.T., Marty-Mazars D., Trick M., Dumas C., Heizmann P. (1999). Pollen-stigma adhesion in *Brassica* spp involves SLG and SLR1 glycoproteins. Plant Cell.

[52] Janson J., Reinders M.C., Valkering A.G.M., Tuyl J.M.V., Keijzer C.J. (1994). Pistil exudate production and pollen tube growth in *Lilium longiflorum* Thunb. Ann. Bot..

[53] Jauh G.Y., Lord E.M. (1995). Movement of the tube cell in the lily style in the absence of the pollen grain and the spent pollen tube. Sex. Plant Reprod..

[54] Lennon K.A., Roy S., Hepler P.K., Lord E.M. (1998). The structure of the transmitting tissue of *Arabidopsis thaliana* (L.) and the path of pollen tube growth. Sex. Plant Reprod..

[55] Mollet J.C., Park S.Y., Nothnagel E.A., Lord E.M. (2000). A lily stylar pectin is necessary for pollen tube adhesion to an in vitro stylar matrix. Plant Cell.

[56] Park S.Y., Jauh G.Y., Mollet J.C., Eckard K.J., Nothnagel E.A., Walling L.L., Lord E.M. (2000). A lipid transfer-like protein is necessary for lily pollen tube adhesion to an in vitro stylar matrix. Plant Cell.

[57] Kim S., Mollet J.C., Dong J., Zhang K., Park S.Y., Lord E.M. (2003). Chemocyanin, a small basic protein from the lily stigma, induces pollen tube chemotropism. Proc. Natl. Acad. Sci. USA.

[58] Kim S.T., Zhang K., Dong J., Lord E.M. (2006). Exogenous free ubiquitin enhances lily pollen tube adhesion to an in vitro stylar matrix and may facilitate endocytosis of SCA. Plant Physiol..

[59] Chae K., Zhang K., Zhang L., Morikis D., Kim S.T., Mollet J.C., De la Rosa N., Tan K., Lord E.M. (2007). Two SCA (stigma/style cysteine-rich adhesin) isoforms show structural differences that correlate with their levels of in vitro pollen tube adhesion activity. J. Biol. Chem..

[60] Chae K., Kieslich C.A., Morikis D., Kim S.C., Lord E.M. (2009). A gain-of-function mutation of *Arabidopsis* lipid transfer protein 5 disturbs pollen tube tip growth and fertilization. Plant Cell.

[61] Chae K., Gonong B.J., Kim S.C., Kieslich C.A., Morikis D., Balasubramanian S., Lord E.M. (2010). A multifaceted study of stigma/style cysteine-rich adhesin (SCA)-like *Arabidopsis* lipid transfer proteins (LTPs) suggests diversified roles for these LTPs in plant growth and reproduction. J. Exp. Bot..

[62] Cheung A.Y., Wang H., Wu H.M. (1995). A floral transmitting tissue-specific glycoprotein attracts pollen tubes and stimulates their growth. Cell.

[63] Wang H., Wu H.M., Cheung A.Y. (1993). Development and pollination regulated accumulation and glycosylation of a stylar transmitting tissue-specific proline-rich protein. Plant Cell.

[64] Rubinstein A.L., Broadwater A.H., Lowrey K.B., Bedinger P.A. (1995). *Pex1*, a pollen-specific gene with an extensin-like domain. Proc. Natl. Acad. Sci. USA.

[65] Michard E., Lima P.T., Borges F., Silva A.C., Portes M.T., Carvalho J.E., Gilliham M., Liu L.H., Obermeyer G., Feijó J.A. (2011). Glutamate receptor-like genes form Ca^2+^ channels in pollen tubes and are regulated by pistil D-serine. Science.

[66] Yu G.H., Zou J., Feng J., Peng X.B., Wu J.Y., Wu Y.L., Palanivelu R., Sun M.X. (2014). Exogenous gamma-aminobutyric acid (GABA) affects pollen tube growth via modulating putative Ca^2+^-permeable membrane channels and is coupled to negative regulation on glutamate decarboxylase. J. Exp. Bot..

[67] Palanivelu R., Brass L., Edlund A.F., Preuss D. (2003). Pollen tube growth and guidance is regulated by *POP2*, an *Arabidopsis* gene that controls GABA levels. Cell.

[68] Franklin-Tong V.E. (1999). Signaling and the modulation of pollen tube growth. Plant Cell.

[69] Kim H.U., Cotter R., Johnson S., Senda M., Dodds P., Kulikauska R., Tang W., Ezcura I., Herzmark P., McCormick S. (2002). New pollen-specific receptor kinases identified in tomato, maize and *Arabidopsis*: The tomato kinases show overlapping but distinct localization patterns on pollen tubes. Plant Mol. Biol..

[70] Muschietti J., Eyal Y., McCormick S. (1998). Pollen tube localization implies a role in pollen-pistil interactions for the tomato receptor-like protein kinases LePRK1 and LePRK2. Plant Cell.

[71] Zhang D., Wengier D., Shuai B., Gui C.P., Muschietti J., McCormick S., Tang W.H. (2008). The pollen receptor kinase LePRK2 mediates growth-promoting signals and positively regulates pollen germination and tube growth. Plant Physiol..

[72] Huang W.J., Liu H.K., McCormick S., Tang W.H. (2014). Tomato pistil factor STIG1 promotes in vivo pollen tube growth by binding to phosphatidylinositol 3-phosphate and the extracellular domain of the pollen receptor kinase LePRK2. Plant Cell.

[73] Wengier D., Valsecchi I., Cabanas M.L., Tang W.H., McCormick S., Muschietti J. (2003). The receptor kinases LePRK1 and LePRK2 associate in pollen and when expressed in yeast, but dissociate in the presence of style extract. Proc. Natl. Acad. Sci. USA.

[74] Marshall E., Costa L.M., Gutierrez-Marcos J. (2011). Cysteine-rich peptides (CRPs) mediate diverse aspects of cell-cell communication in plant reproduction and development. J. Exp. Bot..

[75] Twell D., Wing R., Yamaguchi J., McCormick S. (1989). Isolation and expression of an anther-specific gene from tomato. Mol. Gen. Genet..

[76] Muschietti J., Dircks L., Vancanneyt G., McCormick S. (2010). LAT52 protein is essential for tomato pollen development: Pollen expressing antisense *LAT52* RNA hydrates and germinates abnormally and cannot achieve fertilization. Plant J..

[77] Tang W., Ezcurra I., Muschietti J., McCormick S. (2002). A cysteine-rich extracellular protein, LAT52, interacts with the extracellular domain of the pollen receptor kinase LePRK2. Plant Cell.

[78] Tang W., Kelley D., Ezcurra I., Cotter R., McCormick S. (2010). LeSTIG1, an extracellular binding partner for the pollen receptor kinases LePRK1 and LePRK2, promotes pollen tube growth in vitro. Plant J..

[79] Feiguelman G., Fu Y., Yalovsky S. (2018). ROP GTPases structure-function and signaling pathways. Plant Physiol..

[80] Kaothien P., Ok S.H., Shuai B., Wengier D., Cotter R., Kelley D., Kiriakopolos S., Muschietti J., McCormick S. (2010). Kinase partner protein interacts with the LePRK1 and LePRK2 receptor kinases and plays a role in polarized pollen tube growth. Plant J..

[81] Berken A., Thomas C., Wittinghofer A. (2005). A new family of RhoGEFs activates the Rop molecular switch in plants. Nature.

[82] Shichrur K., Yalovsky S. (2006). Turning on the switch—RhoGEFs in plants. Trends Plant Sci..

[83] Wengier D.L., Mazzella M.A., Salem T.M., McCormick S., Muschietti J.P. (2010). STIL, a peculiar molecule from styles, specifically dephosphorylates the pollen receptor kinase LePRK2 and stimulates pollen tube growth in vitro. BMC Plant Biol..

[84] Chang F., Gu Y., Ma H., Yang Z.B. (2013). AtPRK2 promotes ROP1 activation via RopGEFs in the control of polarized pollen tube growth. Mol. Plant.

[85] Chen L.Y., Shi D.Q., Zhang W.J., Tang Z.S., Liu J., Yang W.C. (2015). The *Arabidopsis* alkaline ceramidase TOD1 is a key turgor pressure regulator in plant cells. Nat. Commun..

[86] Ng C.K., Carr K., McAinsh M.R., Powell B., Hetherington A.M. (2001). Drought-induced guard cell signal transduction involves sphingosine-1-phosphate. Nature.

[87] Coursol S., Fan L.M., Le Stunff H., Spiegel S., Gilroy S., Assmann S.M. (2003). Sphingolipid signalling in *Arabidopsis* guard cells involves heterotrimeric G proteins. Nature.

[88] Wang L., Wang W., Wang Y.Q., Liu Y.Y., Wang J.X., Zhang X.Q., Ye D., Chen L.Q. (2013). *Arabidopsis* galacturonosyltransferase (GAUT) 13 and GAUT14 have redundant functions in pollen tube growth. Mol. Plant.

[89] Ge Z., Bergonci T., Zhao Y., Zou Y., Du S., Liu M.C., Luo X., Ruan H., Garcia-Valencia L.E., Zhong S. (2017). *Arabidopsis* pollen tube integrity and sperm release are regulated by RALF-mediated signaling. Science.

[90] Mecchia M.A., Santos-Fernandez G., Duss N.N., Somoza S.C., Boisson-Dernier A., Gagliardini V., Martinez-Bernardini A., Fabrice T.N., Ringli C., Muschietti J.P. (2017). RALF4/19 peptides interact with LRX proteins to control pollen tube growth in *Arabidopsis*. Science.

[91] Chen L.Y. (2018). Small peptides, big roles—RALFs regulate pollen tube growth and burst in plant reproduction. J. Genet. Genom..

[92] Murphy E., Smet D.I. (2014). Understanding the RALF family: A tale of many species. Trends Plant Sci..

[93] Baumberger N., Ringli C., Keller B. (2001). The chimeric leucine-rich repeat/extensin cell wall protein LRX1 is required for root hair morphogenesis in *Arabidopsis thaliana*. Genes Dev..

[94] Higashiyama T., Kuroiwa H., Kuroiwa T. (2003). Pollen-tube guidance: Beacons from the female gametophyte. Curr. Opin. Plant Biol..

[95] Higashiyama T., Hamamura Y. (2008). Gametophytic pollen tube guidance. Sex. Plant Reprod..

[96] Chae K., Lord E.M. (2011). Pollen tube growth and guidance: Roles of small, secreted proteins. Ann. Bot..

[97] Takeuchi H., Higashiyama T. (2011). Attraction of tip-growing pollen tubes by the female gametophyte. Curr. Opin. Plant Biol..

[98] Higashiyama T., Takeuchi H. (2015). The mechanism and key molecules involved in pollen tube guidance. Annu. Rev. Plant Biol..

[99] Mizukami A.G., Inatsugi R., Jiao J., Kotake T., Kuwata K., Ootani K., Okuda S., Sankaranarayanan S., Sato Y., Maruyama D. (2016). The AMOR arabinogalactan sugar chain induces pollen-tube competency to respond to ovular guidance. Curr. Biol..

[100] Márton M.L., Cordts S., Broadhvest J., Dresselhaus T. (2005). Micropylar pollen tube guidance by egg apparatus 1 of maize. Science.

[101] Okuda S., Tsutsui H., Shiina K., Sprunck S., Takeuchi H., Yui R., Kasahara R.D., Hamamura Y., Mizukami A., Susaki D. (2009). Defensin-like polypeptide LUREs are pollen tube attractants secreted from synergid cells. Nature.

[102] Takeuchi H., Higashiyama T. (2012). A species-specific cluster of defensin-like genes encodes diffusible pollen tube attractants in *Arabidopsis*. PLoS Biol..

[103] Kasahara R.D., Portereiko M.F., Sandaklie-Nikolova L., Rabiger D.S., Drews G.N. (2005). MYB98 is required for pollen tube guidance and synergid cell differentiation in *Arabidopsis*. Plant Cell.

[104] Chen Y.H., Li H.J., Shi D.Q., Yuan L., Liu J., Sreenivasan R., Baskar R., Grossniklaus U., Yang W.C. (2007). The central cell plays a critical role in pollen tube guidance in *Arabidopsis*. Plant Cell.

[105] Li H.J., Zhu S.S., Zhang M.X., Wang T., Liang L., Xue Y., Shi D.Q., Liu J., Yang W.C. (2015). *Arabidopsis* CBP1 is a novel regulator of transcription initiation in central cell-mediated pollen tube guidance. Plant Cell.

[106] Shimizu K.K., Ito T., Ishiguro S., Okada K. (2008). *MAA3* (*MAGATAMA3*) helicase gene is required for female gametophyte development and pollen tube guidance in *Arabidopsis thaliana*. Plant Cell Physiol..

[107] Lu Y.X., Chanroj S., Zulkifli L., Johnson M.A., Uozumi N., Cheung A., Sze H. (2011). Pollen tubes lacking a pair of K^+^ transporters fail to target ovules in *Arabidopsis*. Plant Cell.

[108] Gao Q.F., Gu L.L., Wang H.Q., Fei C.F., Fang X., Hussain J., Sun S.J., Dong J.Y., Liu H.T., Wang Y.F. (2016). Cyclic nucleotide-gated channel 18 is an essential Ca^2+^ channel in pollen tube tips for pollen tube guidance to ovules in *Arabidopsis*. Proc. Natl. Acad. Sci. USA.

[109] Wang T., Liang L., Xue Y., Jia P.F., Chen W., Zhang M.X., Wang Y.C., Li H.J., Yang W.C. (2016). A receptor heteromer mediates the male perception of female attractants in plants. Nature.

[110] Takeuchi H., Higashiyama T. (2016). Tip-localized receptors control pollen tube growth and LURE sensing in *Arabidopsis*. Nature.

[111] Schoenaers S., Balcerowicz D., Costa A., Vissenberg K. (2017). The kinase ERULUS controls pollen tube targeting and growth in *Arabidopsis thaliana*. Front. Plant Sci..

[112] Liu J., Zhong S., Guo X., Hao L., Wei X., Huang Q., Hou Y., Shi J., Wang C., Gu H. (2013). Membrane-bound RLCKs LIP1 and LIP2 are essential male factors controlling male-female attraction in *Arabidopsis*. Curr. Biol..

[113] Higashiyama T., Yabe S., Sasaki N., Nishimura Y., Miyagishima S., Kuroiwa H., Kuroiwa T. (2001). Pollen tube attraction by the synergid cell. Science.

[114] Higashiyama T., Kuroiwa H., Kawano S., Kuroiwa T. (1998). Guidance in vitro of the pollen tube to the naked embryo sac of torenia fournieri. Plant Cell.

[115] Palanivelu R., Preuss D. (2006). Distinct short-range ovule signals attract or repel *Arabidopsis thaliana* pollen tubes in vitro. BMC Plant Biol..

[116] Shimizu K.K., Okada K. (2000). Attractive and repulsive interactions between female and male gametophytes in *Arabidopsis* pollen tube guidance. Development.

[117] Okuda S., Suzuki T., Kanaoka M.M., Mori H., Sasaki N., Higashiyama T. (2013). Acquisition of LURE-binding activity at the pollen tube tip of *Torenia fournieri*. Mol. Plant.

[118] Márton M.L., Fastner A., Uebler S., Dresselhaus T. (2012). Overcoming hybridization barriers by the secretion of the maize pollen tube attractant ZmEA1 from *Arabidopsis* ovules. Curr. Biol..

[119] Schoenaers S., Balcerowicz D., Breen G., Hill K., Zdanio M., Mouille G., Holman T.J., Oh J., Wilson M.H., Nikonorova N. (2018). The auxin-regulated CrRLK1L kinase ERULUS controls cell wall composition during root hair tip growth. Curr. Biol..

[120] Escobar-Restrepo J.M., Huck N., Kessler S., Gagliardini V., Gheyselinck J., Yang W.C., Grossniklaus U. (2007). The FERONIA receptor-like kinase mediates male-female interactions during pollen tube reception. Science.

[121] Hou Y., Guo X., Cyprys P., Zhang Y., Bleckmann A., Cai L., Huang Q., Luo Y., Gu H., Dresselhaus T. (2016). Maternal ENODLs are required for pollen tube reception in *Arabidopsis*. Curr. Biol..

[122] Li C., Yeh F.L., Cheung A.Y., Duan Q., Kita D., Liu M.C., Maman J., Luu E.J., Wu B.W., Gates L. (2015). Glycosylphosphatidylinositol-anchored proteins as chaperones and co-receptors for FERONIA receptor kinase signaling in *Arabidopsis*. Elife.

[123] Gonneau M., Desprez T., Martin M., Doblas V.G., Bacete L., Miart F., Sormani R., Hématy K., Renou J., Landrein B. (2018). Receptor kinase THESEUS1 is a rapid alkalinization factor 34 receptor in *Arabidopsis*. Curr. Biol..

[124] Amien S., Kliwer I., Márton M.L., Debener T., Geiger D., Becker D., Dresselhaus T. (2010). Defensin-like ZmES4 mediates pollen tube burst in maize via opening of the potassium channel KZM1. PLoS Biol..

[125] Liu L., Zheng C., Kuang B., Wei L., Yan L., Wang T. (2016). Receptor-like kinase RUPO interacts with potassium transporters to regulate pollen tube growth and integrity in rice. PLoS Genet..

[126] Liu Y., Joly V., Dorion S., Rivoal J., Matton D.P. (2015). The plant ovule secretome: A different view toward pollen-pistil interactions. J. Proteome Res..

